# Importance of Simultaneous Evaluation of Multiple Risk Factors for Hemodialysis Patients’ Mortality and Development of a Novel Index: Dialysis Outcomes and Practice Patterns Study

**DOI:** 10.1371/journal.pone.0128652

**Published:** 2015-06-01

**Authors:** Eiichiro Kanda, Brian A. Bieber, Ronald L. Pisoni, Bruce M. Robinson, Douglas S. Fuller

**Affiliations:** 1 Department of Nephrology, Tokyo Kyosai Hospital, Meguro, Tokyo, Japan; 2 Life science and bioethics center, Tokyo Medical and Dental University, Bunkyo, Tokyo, Japan; 3 Arbor Research Collaborative for Health, Ann Arbor, MI, United States of America; 4 Department of Internal Medicine, University of Michigan, Ann Arbor, MI, United States of America; Chi-Mei Medical Center, TAIWAN

## Abstract

**Background:**

For hemodialysis (HD) patients, many risk factors for death are associated with each other intricately. However, they are often considered separately in clinical settings. We evaluated the maintenance HD patients’ risk of death within one year from multiple risk factors simultaneously considering their interrelationships using a novel index (survival index, SI) for HD patients in the United States developed using data from the Dialysis Outcomes and Practice Patterns Study (DOPPS).

**Methods:**

We analyzed data from 3899 and 3765 patients to develop and validate SI, respectively. To predict death within one year, candidate models were developed using logistic regression models. The final model was determined by comparing the accuracy among the models for the prediction of deaths.

**Results:**

The model included age; body mass index; serum creatinine, albumin, total cholesterol and phosphorus levels; history of cardiovascular diseases; and arteriovenous fistula use. SI showed a higher accuracy in predicting death (c-statistic, 0.739) than geriatric nutritional risk index (0.647) and serum albumin level (0.637). The probability of death predicted on the basis of SI matched the observed number of deaths. Cox proportional hazard models for time-dependent SI showed that patients with low SI had a higher risk of death than patients with high SI [reference, Group 4 (26.1≤SI)]; Group 1 (SI<12.7), adjusted hazard ratio, 7.97 (95% CI, 5.02, 12.65); Group 2 (12.7≤SI<19.0), 3.18 (95% CI, 1.96, 5.16); Group 3 (19.0≤SI<26.1), 2.20 (95% CI, 1.33, 3.66).

**Conclusion:**

Results of this study suggest that the simultaneous evaluation of multiple risk factors can more accurately assess patients’ prognosis and identify patients at an increased risk of death than single factors.

## Introduction

The risk of death is high in end-stage renal disease (ESRD) patients [1]. Various risk factors for death in hemodialysis (HD) patients have been reported such as age, serum albumin and phosphorus levels, and vascular access [2]. Risk factors are intricately associated with each other. For example, the pathophysiology of malnutrition is characterized by protein-energy wasting (PEW), which leads to atherosclerotic cardiovascular diseases (CVDs), and is a strong risk factor for increased hospitalization and mortality rates in ESRD patients [3–6].

HD patients have some specific characteristics. Serum albumin level is affected by inflammation and age, and does not accurately reflect nutritional status [7]. The association of increased body mass index (BMI) with improved prognosis in HD patients is an example of “reverse epidemiology” [3, 8, 9], and this relationship is modified by HD patients’ characteristics such as age, inflammation, and comorbid conditions [10, 11]. These characteristics make it difficult to evaluate HD patients’ prognosis.

Risk factors in HD patients have often been evaluated separately in clinical settings. To control all risk factors and their complex interrelationships, it is necessary to evaluate them simultaneously. However, to the best of our knowledge, there have been few studies in which the numerically integrated multiple risk factors were evaluated. The purpose of this study was to evaluate the maintenance HD patients’ risk of death within one year considering multiple risk factors and their complex interrelationships. For this purpose, (1) after detection of main risk factors for death within one year using data of HD patients in the Dialysis Outcomes and Practice Patterns Study (DOPPS), a novel index (survival index, SI) was developed. (2) The accuracy of the index to predict death was compared with those of other indices. (3) High-risk patients were identified and their prognosis was evaluated.

## Methods

### Data Source

DOPPS was a worldwide cohort study of in-center HD practice patterns and outcomes in seven countries. The details of the method of DOPPS were reported previously [12, 13]. In brief, there have been phases of DOPPS data collection since the study initiation in 1996. A randomized, stratified selection method was used to identify facilities for participation within each country. Demographic data, cause of ESRD, and mortality data for all HD patients in each facility were collected as cumulative HD census. Detailed patient data within each facility were collected at study entry and at 4-month intervals throughout the study. Institutional review boards approved DOPPS in each facility. Participants gave their informed consent to the use of their clinical records in DOPPS in accordance with the requirements of each review board and facility. Data were collected such that patient anonymity was maintained, and the collected data were anonymized and de-identified prior to analysis. The data were protected by Arbor Research Collaborative for Health, which approved this study.

**Fig 1:**
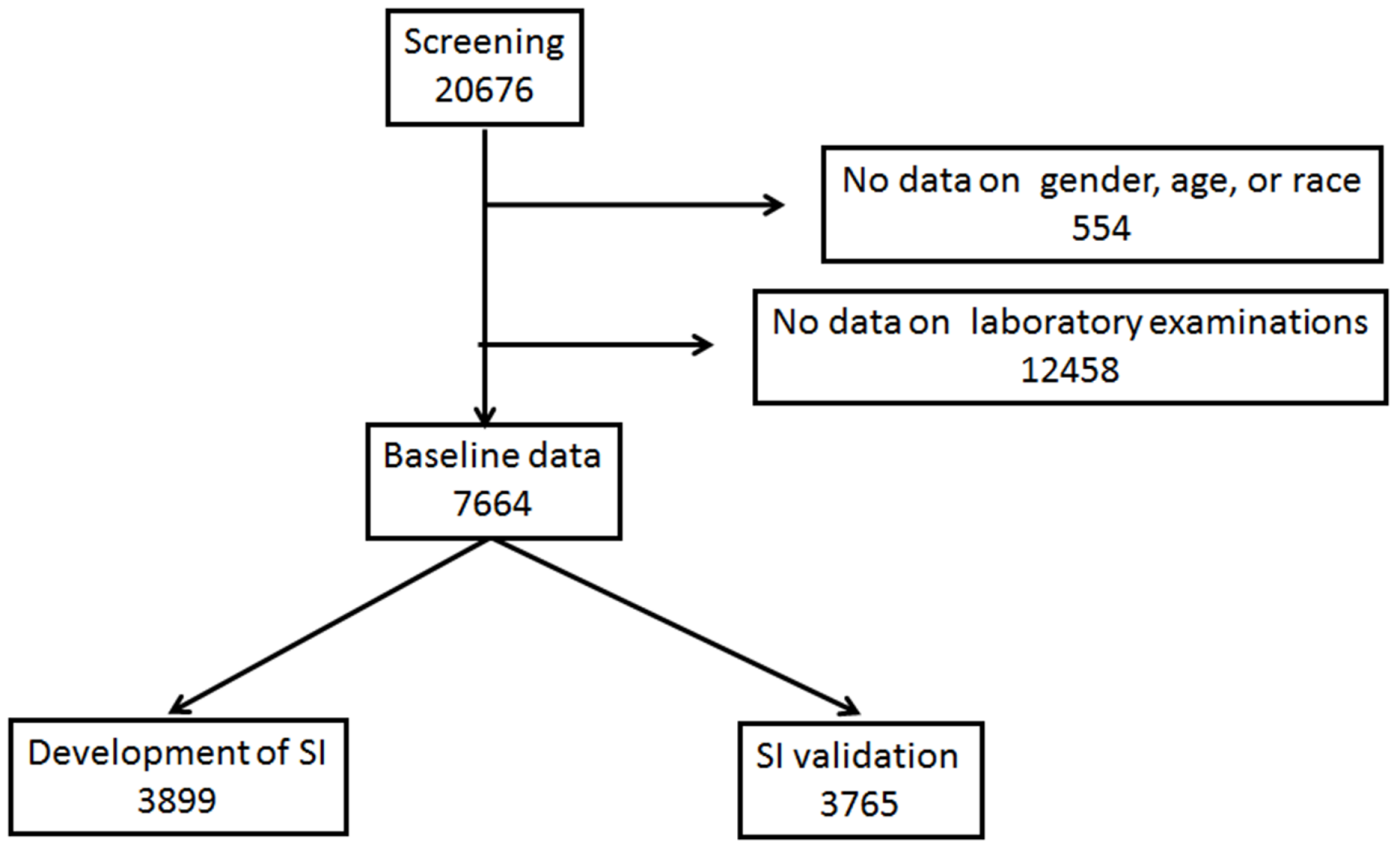
Flow diagram of participants. Abbreviation: SI, survival index.

The United States DOPPS database from phases 1 (1996–2001), 2 (2002–2004), and 3 (2005–2008) was used in this study (Fig 1). The subjects of this study were all HD patients who participated in the United States DOPPS, including incident (vintage <90 days) and maintenance (90 days ≤vintage) HD patients who began dialysis during the study period. Patients were excluded from this study with missing data such as age, gender, race, and laboratory data. The remaining patients were divided randomly into two datasets: a dataset for the development of survival index (SI) and a dataset for SI validation.

Baseline patient data, including age, gender, BMI, race, comorbid conditions [CVDs and hypertension]; diabetes mellitus (DM) as a cause of ESRD; serum creatinine, albumin, glucose, total cholesterol, albumin-corrected calcium, phosphorus, intact parathyroid hormone (PTH), and hemoglobin levels; white blood cell (WBC) count; normalized protein catabolic rate (nPCR); arteriovenous fistula (AVF) use; HD vintage; and Kt/V, were collected from all the patients. The data were followed up every 4 months. BMI was calculated using patients’ postdialysis weight, and laboratory values were obtained from predialysis values. Albumin-corrected calcium level was calculated as [(4—serum albumin level) × 0.8] + serum calcium level [14]. Elevated WBC count, defined as >9000/μl (Yes = 1, No = 0), was a binary variable and treated as a surrogate of inflammation. Controlled hemoglobin level, defined as 10–12 g/dl, was a binary variable. nPCR was determined using the two-point model of urea kinetics developed [15]. Kt/V was a single-pool estimate of dialysis dose. CVDs as comorbid conditions consisted of coronary artery disease, congestive heart failure, cerebrovascular disease, peripheral vascular disease, and other cardiovascular diseases. The ideal BMI for people in the US was assumed to be 22.5 [16]. Geriatric nutritional risk index (GNRI) was calculated using the following formula [17, 18]:

$$\text{GNRI}=14.89\times \text{Alb}+\frac{41.7\times \text{BMI}}{22.5\times {\text{height}}^{2}};$$

for patients where BMI (kg/m^2^) / [22.5×height^2^ (m^2^)] is more than 1,

GNRI=14.89×Alb+41.7 (1)

where BMI is in kg/m^2^; Alb = serum albumin level (g/dl); height is in m^2^.

A modified combined score (CS) was calculated using the following formula [19]:

CS=low serum albumin level score + low BMI score + elevated WBC count (2)

where a low serum albumin level of <3.5g/dl scored Yes = 1, No = 0; a low BMI of <19.6kg/m^2^ was scored Yes = 1, No = 0. Although serum C-related protein (CRP) level was used in the original CS, because there were many missing values of serum CRP levels in our dataset, elevated WBC count was alternatively used in this study.

The outcome was death including all-cause death and cause-specific death (CVD-caused and infection-caused death) within one year. The number of CVD-caused deaths was obtained by summing the number of patients who died of cardiac and cerebral causes. Causes of cardiac death included myocardial infarction, atherosclerotic heart disease, congestive heart failure, cardiomyopathy, arrhythmia, valvular heart disease, pericarditis, and unknown-cause cardiac arrest. Suspected PEW was diagnosed on the basis of International Society of Renal Nutrition & Metabolism (ISRNM) PEW criteria; (1) serum chemistry (serum albumin level <3.8g/dl or serum cholesterol level <100mg/dl), (2) body mass (BMI <23), and (3) dietary intake (nPCR <0.8g/kg/day) [3].

### Statistical methods

Normally distributed variables are presented as mean ± standard deviation (SD); otherwise, the median and interquartile range are presented. Highly skewed variables (e.g., PTH, vintage, and CS) were transformed with the natural logarithm function prior to use in models [ln(PTH), ln(vintage), ln(CS)]. Intergroup comparisons were performed using the chi-square test, t-test, Mann-Whitney U-test, one-way analysis of variance, and Kruskal-Wallis test as appropriate.

In the dataset for the development of SI, candidate models for SI were developed using multivariate logistic regression models as follows: where P(X) = probability of all-cause death,

$$\begin{array}{l} \text{SI}=-10\times \left[\text{Logit P}\left(\text{X}\right)\right]\\ =-10\times \text{ln}\left(\frac{\text{P}\left(\text{X}\right)}{1-\text{P}\left(\text{X}\right)}\right)\\ =-10\times \left(\hat{\beta_{0}}+\text{Σ}\hat{\beta_{i}}\hat{{\text{X}}_{i}}\right) \end{array}$$ (3)

The coefficients were rounded. Candidate variables were selected for inclusion in the SI model based on previous reports, and the results of analysis using univariate logistic regression models (*p*<0.1) [10, 11, 20]. The candidate variables were as follows: age; gender; BMI; square of BMI; race; CVDs; hypertension; DM; serum creatinine, albumin, total cholesterol and phosphorus levels; square of serum phosphorus level; elevated WBC count; controlled hemoglobin level; nPCR; AVF use; ln(vintage); and Kt/V. The square of BMI and serum phosphorus level were listed as candidate variables for evaluating the U-shaped relationship between each variable and HD patients’ mortality. The interactions among BMI, the square of BMI, age, and elevated WBC count were evaluated [10, 11]. Albumin-corrected calcium level and ln(PTH) were not included in the models, because there were their collinearities with serum phosphorus level, and serum phosphorus level is more associated with all-cause mortality than serum calcium and PTH levels in a systematic review [21]. Candidate models were constructed using the hierarchical backward elimination procedure: (1) the initial model was constructed as hierarchically well formulated, and (2) the interaction variables and confounders were statistically evaluated (*p*<0.05). Next, we constructed logistic regression models derived from the initial model by decreasing the variables to find a model that was more easily calculable than the initial model. Considering their c-statistics, Akaike information criterion (AIC), and the results of Hosmer-Lemeshow (HL) goodness-of-fit tests, several models were selected as candidate models for SI. Then, in a dataset for the SI validation, the final model was determined using the models’ c-statistics, AICs, and the result of HL tests for the prediction of all-cause, CVD-caused, and infection-caused deaths within one year. The receiver operating characteristic (ROC) curves of SI for the prediction of all-cause death within one year were used to compare with those of other indices. To investigate the relationship between SI and various patient characteristic factors, Pearson’s correlation coefficients were examined. The predicted probability of all-cause death occurring in each patient was predicted using the following formula:

$$\text{Predicted probability P}\left(\text{X}\right)=\frac{1}{1+\text{exp}\left(\text{SI}/10\right)}$$ (4)

The patients were divided into four groups on the basis of the quartile cut points of SI. Patients’ survival curves were derived by Kaplan-Meier analysis. Cox proportional hazards models (PHMs) were used to compare the risk of the outcome for baseline SI between groups. Then, SI was treated as a time-dependent variable. Cox PHMs for treating SI as a time-dependent covariate were also examined. Adjusted variables in Cox PHMs and time-dependent Cox PHMs were the baseline characteristics including gender, race, hypertension, DM, serum albumin-corrected calcium levels, hemoglobin level, ln(PTH), elevated WBC count, nPCR, ln(vintage), and Kt/V. The results are presented here as hazard ratios (HR) with 95% confidence interval (CI). To keep the loss of data minimum, if a patient’s SI just before the primary outcome was missing, the last SI was used in the time-dependent Cox PHMs. Statistical significance was defined as *p*<0.05. These analyses were conducted using SAS, version 9.2 (SAS, Inc., Cary, North Carolina).

## Results

### Baseline characteristics

The study population consisted of 7664 patients (Fig 1). They were randomly assigned into two groups; one group for SI development and the other for SI validation (Fig 1). No significant differences in patient characteristics between the development and validation datasets were observed (Table 1).

**Table 1:** Baseline and demographic characteristics in datasets.

	All	SI development	SI validation	*p*
N (%)	7664	3899 (50.9)	3765 (49.1)	
Male (%)	4133 (53.9)	2100 (53.9)	2033 (54.0)	0.90
Age (years)	61±16	62±16	61±16	0.77
BMI (kg/m^2^)	26.3±6.8	26.3±6.8	26.3±6.7	0.73
Race (%)				0.78
White (%)	4306 (56.2)	2200 (56.4)	2106 (55.9)	
Black (%)	2677 (34.9)	1348 (34.6)	1329 (35.3)	
Other races (%)	681 (8.9)	351 (9.0)	330 (8.8)	
CVDs (%)	5819 (75.9)	2974 (76.3)	2845 (75.6)	0.47
Hypertension (%)	6597 (86.1)	3356 (86.1)	3241 (86.1)	0.76
DM (%)	2731 (35.6)	1408 (36.1)	1323 (35.1)	0.37
Creatinine (mg/dl)	8.6±3.5	8.6±3.5	8.6±3.5	0.99
Albumin (g/dl)	3.6±0.5	3.6±0.5	3.6±0.5	0.71
Glucose (mg/dl)	140.2±71.7	140.4±68.3	140.0±75.2	0.88
Total cholesterol (mg/dl)	166.1±45.6	165.3±45.2	167.0±46.1	0.11
Albumin-corrected calcium (mg/dl)	9.2±1.0	9.2±1.0	9.2±1.0	0.74
Phosphorus (mg/dl)	5.7±1.9	5.7±1.8	5.7±1.9	0.89
Intact PTH (pg/mL)	352.7±471.3, 216 (93, 438)	365.5±474.1, 219.7 (94, 442)	348.8±468.3, 212 (91.3, 433)	0.21
White blood cell count (1000/μl)	7.7±2.9	7.6±2.8	7.7±2.9	0.16
Hemoglobin (g/dl)	10.8±1.7	10.8±1.6	10.9±1.7	0.75
nPCR	0.96±0.26	0.96±0.26	0.96±0.26	0.72
GNRI	94.4±8.5	94.3±8.3	94.5±8.7	0.40
CS	0.67±0.75, 1 (0, 1)	0.66±0.75, 1 (0, 1)	0.67±0.76, 1 (0, 1)	0.75
AVF (%)	1752 (22.9)	885 (22.7)	867 (23.0)	0.66
Vintage (years)	2.24±3.38, 0.92 (0.03, 3.12)	2.2±3.3, 0.90 (0.03, 3.10)	2.28±3.44, 0.94 (0.03, 3.15)	0.79
Kt/V	1.37±0.34	1.37±0.34	1.37±0.34	0.79
All death (%)	1138 (14.8)	589 (15.1)	549 (14.6)	0.52
CVD-caused death (%)	536 (7.0)	282 (7.2)	254 (6.7)	
Infection-caused death (%)	255 (3.3)	131 (3.4)	124 (3.3)	
Other-caused death (%)	347 (4.5)	176 (4.5)	171 (4.5)	
Hospitalization (%)	4551 (59.4)	2322 (59.6)	2229 (59.2)	0.75
PEW (%)	401 (5.2)	207 (5.3)	194 (5.2)	0.63

No statistical differences in baseline and demographic characteristics between datasets were observed. Values are expressed as mean ± standard deviation. Serum intact PTH level, vintage and follow-up days are also shown as median and interquartile range. The values were compared between the datasets by the chi-square test, t-test, or Mann-Whitney U-test as appropriate.

Abbreviations: SI, Survival index; BMI, body mass index; CVDs, cardiovascular diseases as comorbid conditions; DM, diabetes mellitus as a cause of end-stage renal disease; PTH, parathyroid hormone; nPCR, normalized protein catabolic rate; GNRI, geriatric nutritional risk index; CS, Combined score; AVF, arteriovenous fistula use; PEW, protein energy wasting.

### Development of candidate models for SI

The initial model did not contain the following variables: gender (*p* = 0.54), race (*p* = 0.17), DM (*p* = 0.94), controlled hemoglobin level (*p* = 0.69), nPCR (*p* = 0.30), ln(vintage) (*p* = 0.27) and Kt/V (*p* = 0.22). Elevated WBC count; the squares of BMI and serum phosphorus level; and the variables that represented interactions were evaluated, but not retained in Model 1. We evaluated additional models containing subsets of the covariates retained in Model 1. Models 1, 3, 6, and 11 were selected as candidate models for SI, because their c-statistics for the prediction of all-cause death were higher than other models (Table 2).

**Table 2:** Comparison of SI candidate models.

Model	Variables	Number of variables	C-statistic	AIC	HL test (*p*)
1	Age, BMI, Cr, Alb, Tchol, P, CVDs, HT, AVF	9	0.730	3296	0.95
2	Age, BMI, Cr, Alb, Tchol, P, CVDs, HT	8	0.726	3296	0.45
3	Age, BMI, Cr, Alb, Tchol, P, CVDs, AVF	8	0.728	3296	0.65
4	Age, BMI, Cr, Alb, Tchol, P, HT, AVF	8	0.725	3296	0.54
5	Age, BMI, Cr, Alb, Tchol, CVDs, HT, AVF	8	0.726	3296	0.99
6	Age, BMI, Cr, Alb, P, CVDs, HT, AVF	8	0.729	3296	0.23
7	Age, BMI, Cr, Tchol, P, CVDs, HT, AVF	8	0.717	3296	0.11
8	Age, BMI, Alb, Tchol, P, CVDs, HT, AVF	8	0.725	3296	0.93
9	Age, Cr, Alb, Tchol, P, CVDs, HT, AVF	8	0.722	3296	0.83
10	BMI, Cr, Alb, Tchol, P, CVDs, HT, AVF	8	0.692	3296	0.06
11	Age, BMI, Cr, Alb, P, CVDs, HT, AVF	7	0.727	3296	0.15

Models 1, 3, 6, and 11 were selected as candidate models for SI.

Abbreviations: SI, Survival index; AIC, Akaike information criterion; HL test, Hosmer-Lemeshow test; BMI, body mass index; Cr, serum creatinine level; Alb, serum albumin level; Tchol, serum total cholesterol level; P, serum phosphorus level; CVDs, cardiovascular diseases as comorbid conditions; HT, hypertension; AVF, arteriovenous fistula use.

Using the dataset for the SI validation, we compared the capability of candidate models to predict all-cause death within one year. Model 3 showed the highest c-statistics for all-cause death and CVD-caused death (Table 3). Model 3 also showed an adequate fit to the data, as determined by the HL test for all-cause death. Therefore, Model 3 was selected for SI. The formula for SI was as follows:

SI = 10−(0.4×Age)+(0.3×BMI)+(0.7×Cr)+(6×Alb)+(0.03×Tchol) −(P)−(2×CVDs)+(2×AVF) (5)

where Age is in years; BMI is in kg/m^2^; Cr = serum creatinine level (mg/dl); Alb = serum albumin level (g/dl); Tchol = serum total cholesterol level (mg/dl); P = serum phosphorus level (mg/dl); CVDs = cardiovascular diseases as comorbid conditions, Yes = 1, No = 0; AVF = arteriovenous fistula use, Yes = 1, No = 0.

**Table 3:** Comparison of the prediction of death between candidate SI models using the dataset for SI validation.

	All death			CVD-caused death			Infection-caused death		
Model	C-statistic	AIC	HL test (*p*)	C-statistic	AIC	HL test (*p*)	C-statistic	AIC	HL test (*p*)
1	0.737	3100	0.49	0.714	1842	0.49	0.791	575	0.17
3	0.739	3129	0.62	0.718	1862	0.92	0.777	584	0.54
6	0.734	3100	0.06	0.708	1843	0.70	0.781	575	0.69
11	0.736	2805	0.50	0.712	1862	0.54	0.764	584	0.56
GNRI	0.647	3129	0.04	0.606	1862	0.53	0.663	584	0.06
CS	0.607	3130	0.95	0.576	1862	0.48	0.569	584	0.34
Age	0.675	3129	0.14	0.671	1862	0.90	0.577	584	0.71
BMI	0.608	3129	0.84	0.585	1862	0.11	0.541	584	0.37
Cr	0.642	3129	0.05	0.625	1862	0.10	0.512	584	0.35
Alb	0.637	3129	0.02	0.607	1862	0.54	0.646	584	0.56

Model 3 was selected for SI, because it showed the highest c-statistic for the prediction of all-cause death.

Abbreviations: SI, Survival index; all death, all-cause death; CVD, cardiovascular disease; PEW, protein energy wasting; AIC, Akaike information criterion; HL test, Hosmer-Lemeshow test; GNRI, geriatric nutritional risk index; CS, Combined score; BMI, body mass index; Cr, serum creatinine level; Alb, serum albumin level.

**Fig 2:**
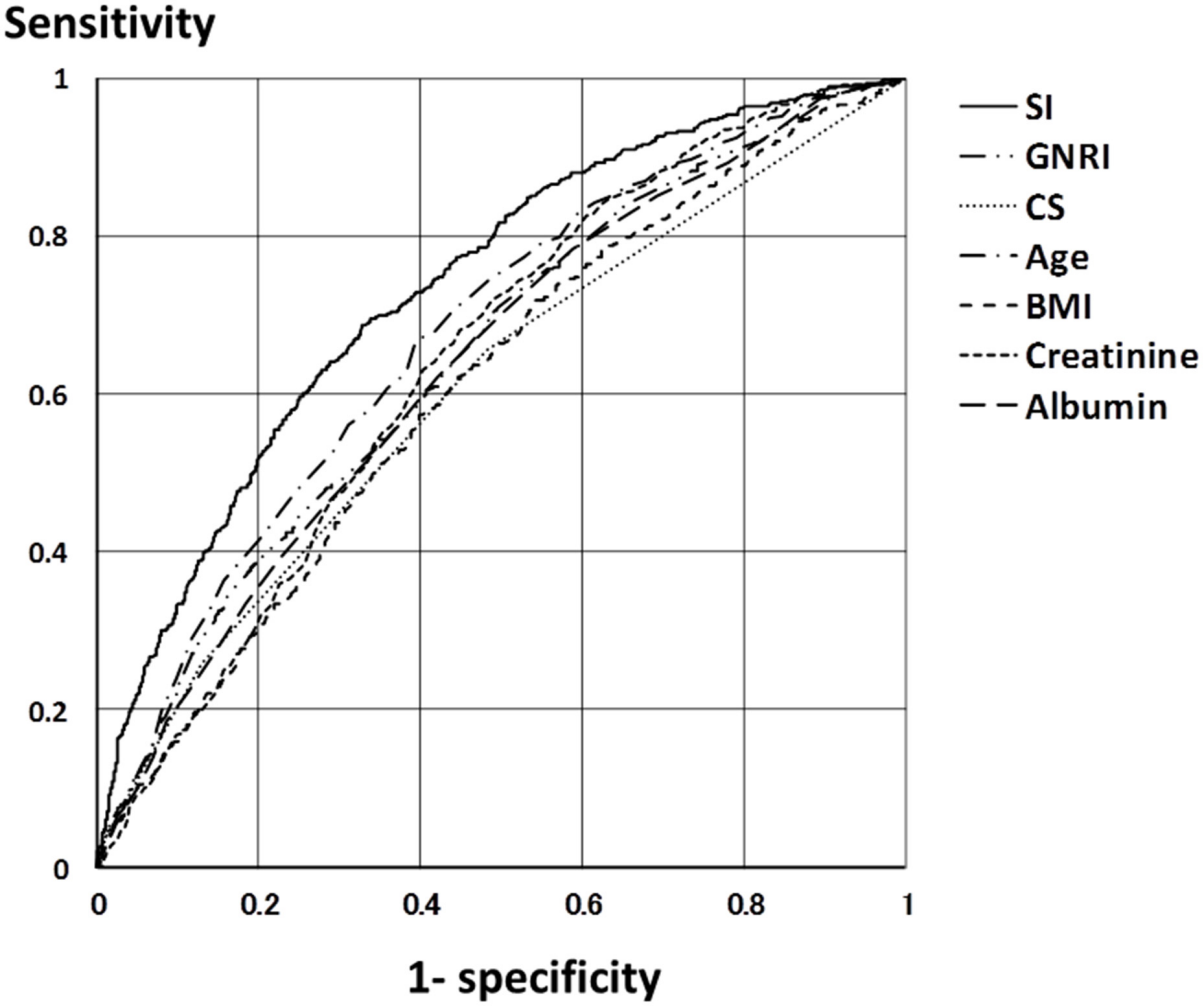
Receiver operating characteristic curves of SI and other indices for prediction of all-cause death within one year. SI had the highest area under the receiver operating characteristic curve for all-cause death than other indices. Abbreviations: SI, survival index; GNRI, geriatric nutritional risk index; CS, combined score; BMI, body mass index; creatinine, serum creatinine level; albumin, serum albumin level.

SI (Model 3) showed higher c-statistics for all-cause death, CVD-caused death, infection-caused death, hospitalization and PEW than GNRI, CS and single indices (Tables 3 and 4) (Fig 2). Age showed higher c-statistics for all-cause death and CVD-caused death than GNRI, CS, BMI, serum creatinine and albumin levels. Serum creatinine and albumin levels showed higher c-statistics for hospitalization than GNRI, CS, age and BMI. BMI showed higher c-statistics for PEW than GNRI, CS, age, serum creatinine and albumin levels.

**Table 4:** Comparison of the prediction of death between candidate SI models using the dataset for SI validation.

	Hospitalization			PEW		
Model	C-statistic	AIC	HL test (*p*)	C-statistic	AIC	HL test (*p*)
1	0.588	5051	0.29	0.915	1432	0.003
3	0.581	5093	0.66	0.915	1433	0.009
6	0.588	5051	0.48	0.915	1432	0.002
11	0.581	5093	0.61	0.915	1434	0.001
GNRI	0.538	5093	0.013	0.848	1434	0.0001
CS	0.520	5093	0.63	0.719	1434	0.025
Age	0.525	5093	0.17	0.606	1434	0.25
BMI	0.513	5093	0.43	0.851	1434	0.0001
Cr	0.549	5093	0.38	0.711	1434	0.52
Alb	0.546	5093	0.34	0.797	1434	0.0001

Model 3 was selected for SI.

Abbreviations: SI, Survival index; all death, all-cause death; CVD, cardiovascular disease; PEW, protein energy wasting; AIC, Akaike information criterion; HL test, Hosmer-Lemeshow test; GNRI, geriatric nutritional risk index; CS, Combined score; BMI, body mass index; Cr, serum creatinine level; Alb, serum albumin level.

### Characteristics of SI

**Fig 3:**
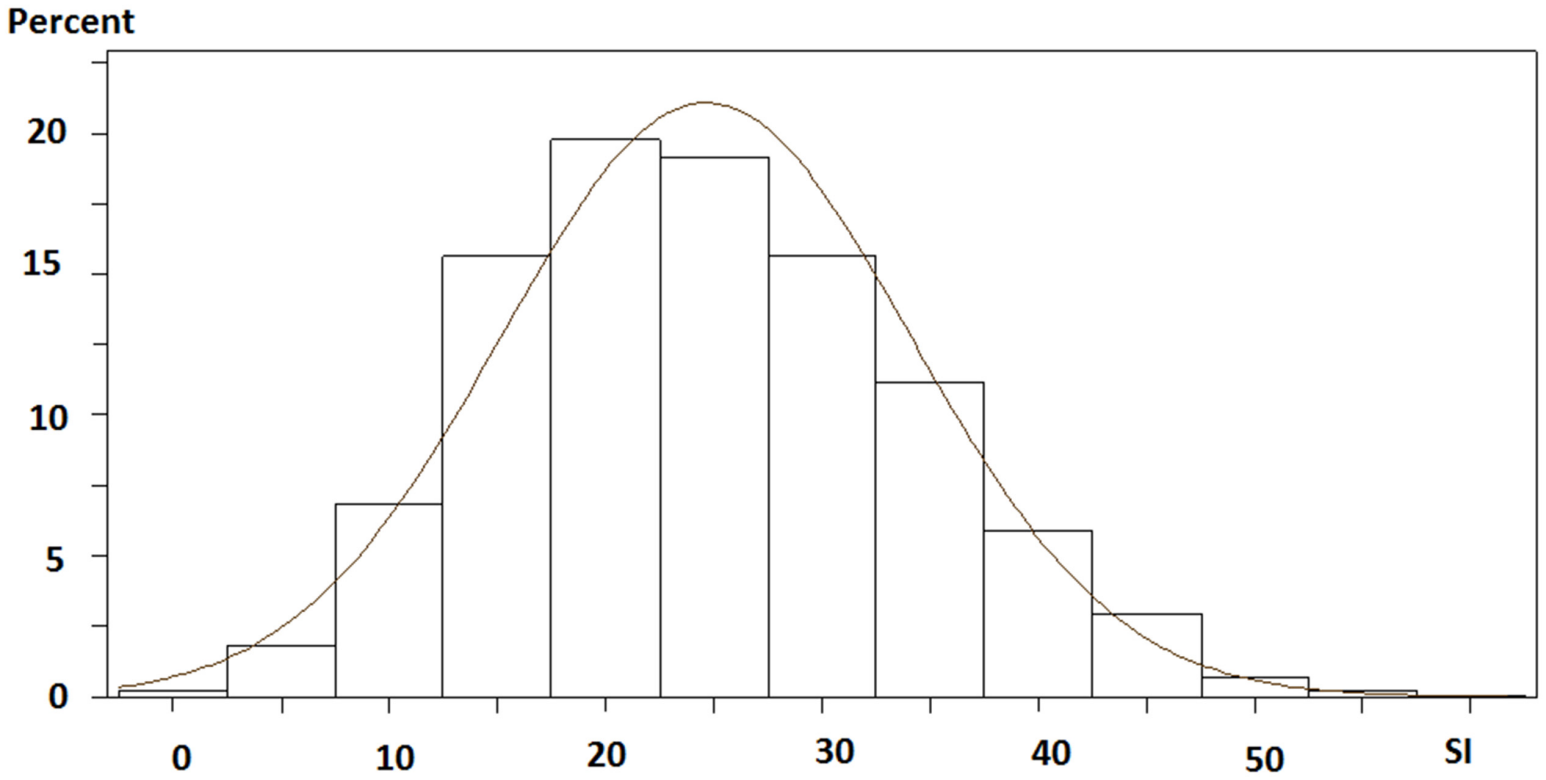
Histogram of SI. Abbreviations: SI, Survival index.

The characteristics of SI were examined using the validation dataset. The mean SI ± SD was 19.6 ± 9.5, with quartile cut points of 12.7, 19.0, and 26.1 (Fig 3). And SI was associated with albumin-corrected calcium level (r = -0.056, *p* = 0.0006), ln(PTH) (r = 0.13, *p* = 0.0001), hemoglobin level (r = 0.077, *p* = 0.0001), nPCR (r = 0.14, *p* = 0.0001), GNRI (r = 0.52, *p* = 0.0001), ln(vintage) (r = 0.26, *p* = 0.0001), ln(CS) (r = -0.26, *p* = 0.0001), and Kt/V (r = -0.058, *p* = 0.001).

**Fig 4:**
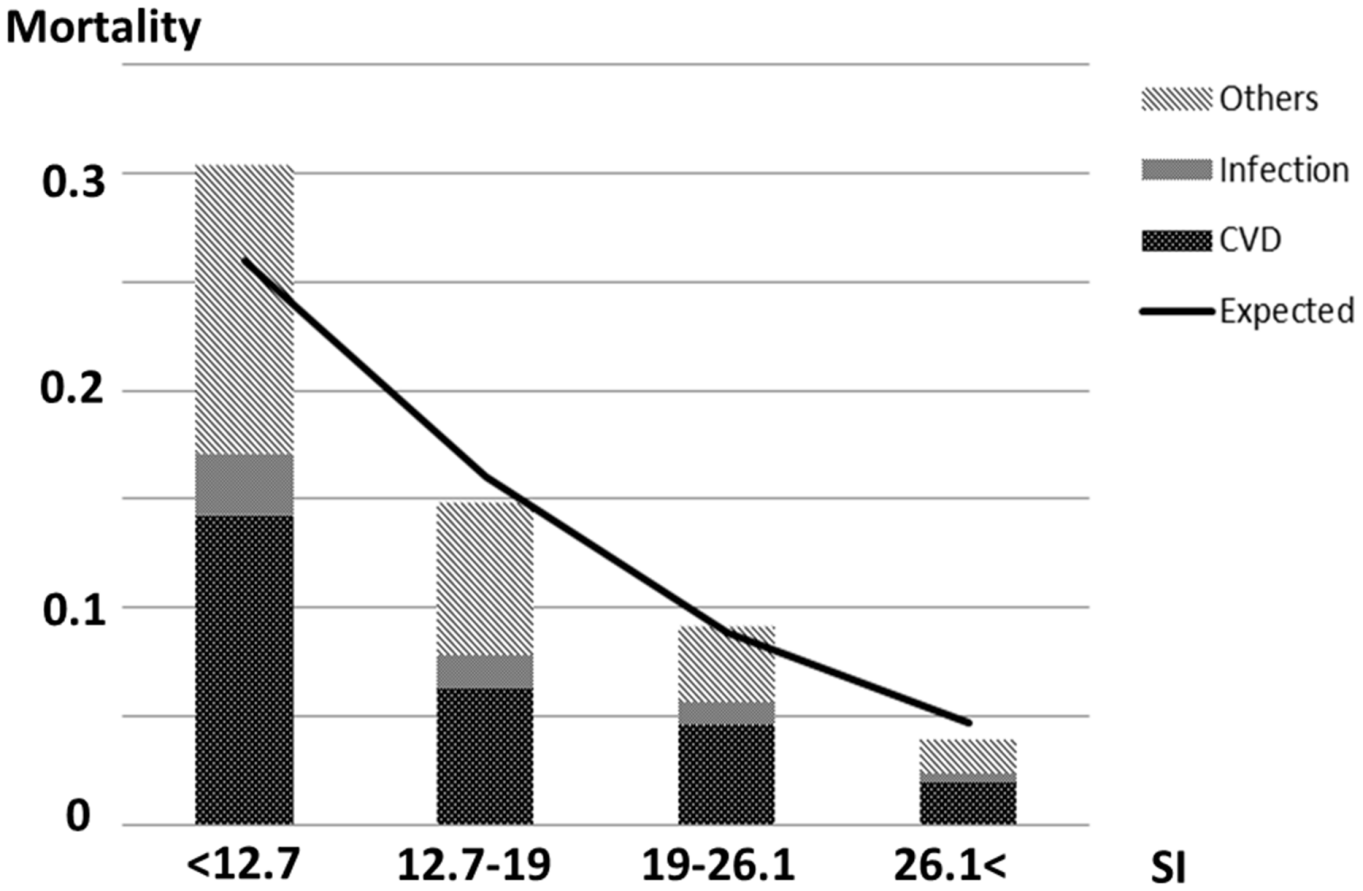
Observed vs predicted incidence of death within one year among SI quartiles. The bar graphs show the observed incidence of all-cause deaths (CVD-caused, infection-caused, and other-caused death). The line graph shows the incidence of deaths predicted using SI. The observed incidences are in good agreement with the predicted incidence. Abbreviations: SI, Survival index; CVD, cardiovascular disease-caused death; infection, infection-caused death.

The predicted probability of death was shown in Table 5. We compared the probability of death predicted on the basis of SI quartiles with the observed number of deaths (Fig 4). The probability and observed number of deaths similarly tended to decrease with increasing SI.

**Table 5:** Predicted probability and SI.

SI	Predicted probability
-4	0.60
-2	0.55
0	0.50
2	0.45
4	0.40
6	0.35
8	0.31
10	0.27
12	0.23
14	0.20
16	0.17
18	0.14
20	0.12
22	0.10
24	0.08
26	0.07
28	0.06
30	0.05
35	0.03
40	0.02
50	0.01

Predicted probability was calculated using the following formula:
$$\text{P}\left(\text{X}\right)=\frac{1}{1+\text{exp}\left(\text{SI}/10\right)}$$
Abbreviations: SI, Survival index; predicted probability, predicted probability of all-cause death.

The c-statistics of SI for all-cause death were compared with those of other indices on the basis of the stratification of patients’ characteristics. The c-statistics of SI in incident and maintenance HD patients were 0.743 and 0.730, respectively, and higher than other indices: GNRI (0.625, 0.648), CS (0.558, 0.615); age (0.687, 0.669), BMI (0.624, 0.602), serum creatinine level (0.600, 0.655), and serum albumin level (0.616, 0.640). The c-statistics of SI in patients with DM as a cause of ESRD and non-DM were 0.701 and 0.751, respectively, which were also higher than other indices: GNRI (0.580, 0.682); CS (0.573, 0.626); age (0.648, 0.685), BMI (0.642, 0.592), serum creatinine level (0.584, 0.675), and serum albumin level (0.564, 0.676).

### Risk of death and SI

In the validation dataset, significant differences in the distribution of baseline characteristics were observed between the groups: Group 1, SI<12.7; Group 2, 12.7≤SI<19.0; Group 3, 19.0≤SI<26.1; and Group 4, 26.1≤SI (Table 6). In Group 1, the number of patients who died was higher than those in the other groups.

**Table 6:** Baseline and demographic characteristics in validation dataset.

	Group 1	Group 2	Group 3	Group 4	*p*
N (%)	949 (25.2)	933 (24.8)	940 (25.0)	943 (25.0)	
Male (%)	481 (50.7)	489 (52.4)	487 (51.8)	576 (61.1)	0.0001
SI	8.2±3.5	15.8±1.8	22.4±2.0	32.2±4.9	0.0001
Age (years)	77±8	68±9	57±10	43±11	0.0001
BMI (kg/m^2^)	23.3±4.4	25.6±5.1	27.5±6.6	29.0±8.6	0.0001
Race (%)					0.0001
White (%)	689 (72.6)	579 (62.1)	477 (50.7)	361 (38.3)	
Black (%)	187 (19.7)	279 (29.9)	373 (39.7)	490 (52.0)	
Other races (%)	73 (7.7)	75 (8.0)	90 (9.6)	92 (9.7)	
CVDs (%)	854 (90.0)	782 (83.8)	713 (75.9)	496 (52.6)	0.0001
Hypertension (%)	810 (85.4)	813 (87.1)	811 (86.3)	807 (85.6)	0.52
DM (%)	318 (33.5)	399 (42.8)	409 (43.5)	197 (20.9)	0.0001
Creatinine (mg/dl)	6.3±2.3	7.6±2.5	8.9±2.8	11.7±3.8	0.0001
Albumin (g/dl)	3.3±0.5	3.6±0.5	3.7±0.5	4.0±0.5	0.0001
Glucose (mg/dl)	135.1±63.4	145.9±73.8	153.7±86.3	125.3±73.4	0.0001
Total cholesterol (mg/dl)	151.7±39.9	168.8±40.5	175.6±48.1	172.0±51.3	0.0001
Albumin-corrected calcium (mg/dl)	9.3±0.9	9.3±0.9	9.3±1.0	9.2±1.1	0.019
Phosphorus (mg/dl)	5.2±1.8	5.5±1.7	5.8±2.0	6.1±2.1	0.0001
Intact PTH (pg/mL)	278.5±360.1, 170 (76, 334)	315.1±415.2, 200.5 (88.5, 396.5)	334.2±406.7, 208 (91, 416)	465.2±622.3, 280.5 (115, 586)	0.0001
White blood cell (1000/μl)	8.2±3.2	7.9±3.0	7.7±2.8	7.2±2.6	0.0001
Hemoglobin (g/dl)	10.6±1.6	10.8±1.6	10.7±1.7	11.0±1.8	0.0001
nPCR	0.90±0.27	0.95±0.26	0.98±0.26	1.00±0.25	0.0001
GNRI	88.2±8.6	94.1±7.4	96.1±7.2	99.6±7.2	0.0001
CS	1.09±0.82, 1 (0, 2)	0.66±0.75, 1 (0, 1)	0.54±0.68, 0 (0, 1)	0.39±0.58 0 (0, 1)	0.0001
AVF (%)	101 (10.6)	196 (21.0)	225 (23.9)	345 (36.6)	0.0001
Vintage (years)	1.21±2.13, 0.17 (0.02, 1.61)	2.06±3.12, 0.87 (0.03, 2.74)	2.40±3.40, 1.18 (0.04, 3.37)	3.43±4.36, 1.93 (0.28, 4.82)	0.0001
Kt/V	1.38±0.35	1.39±0.35	1.36±0.33	1.34±0.32	0.009
All death (%)	288 (30.4)	138 (14.8)	86 (9.2)	37 (3.9)	0.0001
CVD-caused death (%)	135 (14.2)	58 (6.2)	43 (4.6)	18 (1.9)	
Infection-caused death (%)	41 (4.3)	40 (4.3)	27 (2.9)	16 (1.7)	
Other-caused death (%)	112 (11.8)	40 (4.3)	16 (1.7)	3 (0.3)	
Hospitalization	615 (64.8)	562 (60.2)	558 (59.4)	494 (52.4)	0.0001
PEW (%)	117 (12.3)	45 (4.8)	20 (2.1)	12 (1.3)	0.0001

The numbers of deaths, frequency of hospitalization and PEW were higher in Group 1 than in the other groups. Values are expressed as mean ± standard deviation. Serum intact PTH level and vintage are presented as median and interquartile range. The values were compared between the datasets by the chi-square test, one-way analysis of variance, or Kruskal-Wallis test as appropriate. Patients were categorized into four groups on the basis of quartiles of SI.

Abbreviations: Group 1, SI<12.7; Group 2, 12.7≤SI<19.0; Group 3, 19.0≤SI<26.1; Group 4, 26.1≤SI; SI, Survival index; BMI, body mass index; CVDs, cardiovascular diseases as comorbid conditions; DM, diabetes mellitus as a cause of end-stage renal disease; PTH, intact parathyroid hormone; nPCR, normalized protein catabolic rate; GNRI, geriatric nutritional risk index; CS, combined score; AVF, arteriovenous fistula use; PEW, protein energy wasting.

**Fig 5:**
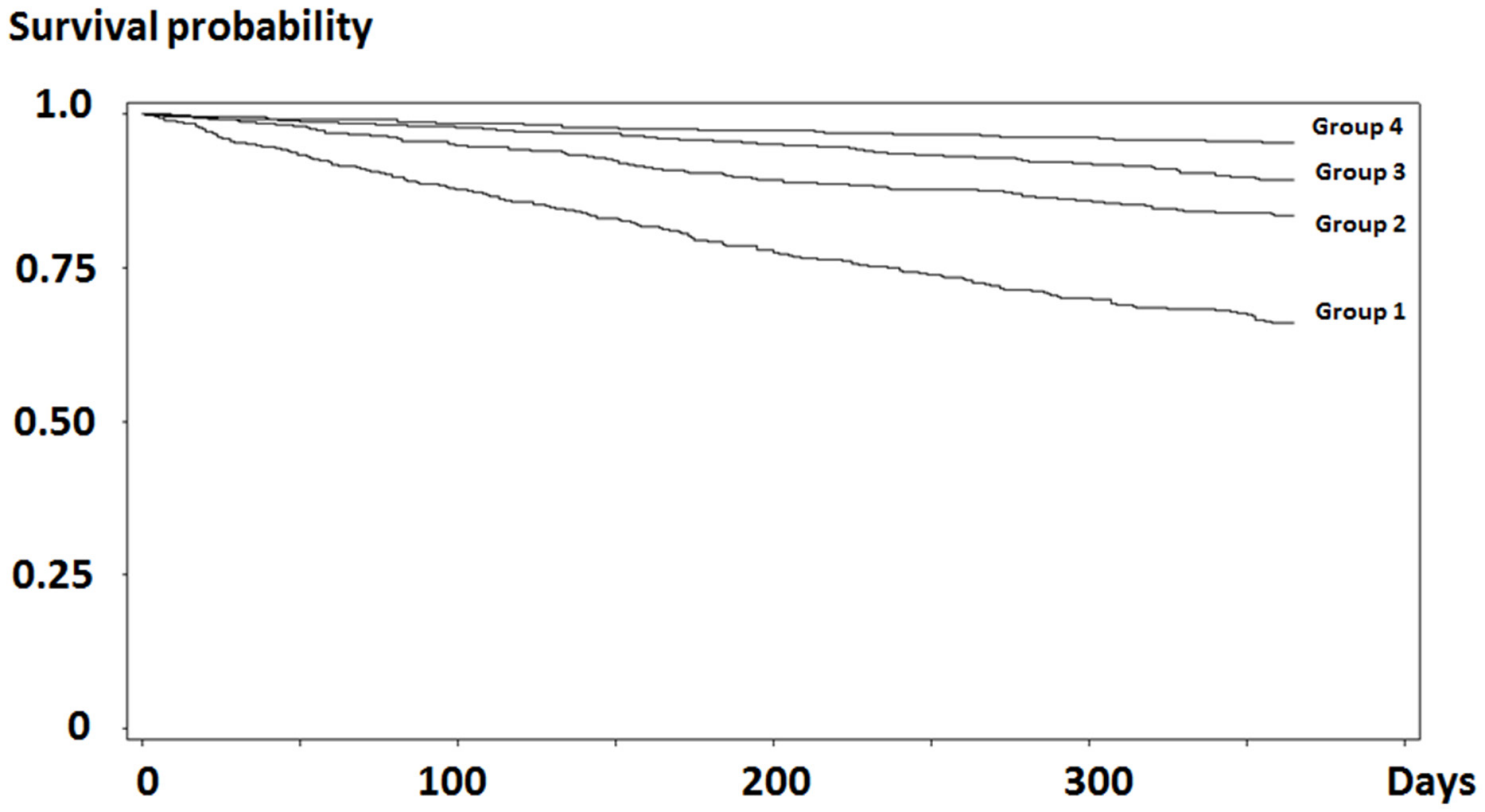
Association between SI and mortality. The Kaplan-Meier survival curve of Group 1 was compared with those of the other groups. The Group 1 showed the lowest survival probability. Abbreviations: SI, survival index; Group 1, SI<12.7; Group 2, 12.7≤SI<19.0; Group 3, 19.0≤SI<26.1; Group 4, 26.1≤SI.

Kaplan-Meier analysis showed that the Group 1 had a higher mortality rate than the other groups [log-rank and Wilcoxon tests, *p* = 0.0001 (Fig 5)]. Cox PHMs and adjusted Cox PHMs showed that a 1 unit increase in SI decreased the risk of death, and that Group 1 showed a high risk of death (reference, Group 4). Analysis of SI as a time-updated effect also showed similar tends (Table 7).

**Table 7:** Hazard ratio for death and SI.

	Unadjusted Cox PHM	Adjusted Cox PHM	Unadjusted time-dependent Cox PHMs	Adjusted time-dependent Cox PHMs
SI (1unit increase)	0.91 (0.90, 0.92)	0.91 (0.89, 0.92)	0.91 (0.90, 0.92)	0.90 (0.89, 0.92)
SI Groups				
Group 1	8.86 (6.29, 12.48)	9.38 (5.91, 14.88)	9.04 (6.28, 13.02)	7.97 (5.02, 12.65)
Group 2	3.80 (2.65, 5.46)	3.32 (2.07, 5.32)	4.32 (2.95, 6.33)	3.18 (1.96, 5.16)
Group 3	2.30 (1.57, 3.39)	2.55 (1.57, 4.14)	2.48 (1.64, 3.74)	2.20 (1.33, 3.66)
Group 4	Reference	Reference	Reference	Reference

Values are given as HRs (95% CI). The Cox proportional hazards models and the time-dependent Cox models were adjusted for the baseline characteristics including gender, race, hypertension, DM, serum albumin-corrected calcium level, hemoglobin level, ln(PTH), elevated WBC count, nPCR, ln(vintage), and Kt/V. Patients were categorized into four groups on the basis of quartiles of SI. In the analysis using time-dependent Cox models, groups were treated as time-dependent variables. The *p* values of all results were less than 0.05.

Abbreviations: SI, Survival index; HR, hazard ratio; CI, confidence interval; DM, diabetes mellitus as a cause of end-stage renal disease; PTH, intact parathyroid hormone; WBC, white blood cell; nPCR, normalized protein catabolic rate.

## Discussion

We were able to develop SI after detection of main risk factors for death within one year, and evaluate precisely the HD patients’ prognosis. SI included various risk factors for death including their interrelationships, which were consistent with previous reports [2, 3, 22]. SI showed a higher accuracy in identifying high-risk patients than other single indices. High-risk patients were identified from the view point of multiple risk factors using SI.

The prediction of all-cause death of HD patients based on SI was more accurate than that based on SI’s individual components. It has been reported that the combination of low BMI and low serum albumin and creatinine levels reflects a high risk of death more accurately than each factor [22]. On the other hand, the combination of serum albumin and CRP levels and BMI predicts HD patients’ mortality more accurately than these indices [19]. These findings suggest that an index including multiple variables more accurately predicts HD patients’ mortality than single indices. It has been reported that achieving multiple therapeutic targets is associated with better survival in HD patients than achieving fewer targets [23, 24]. These studies indicate the importance of an index for simultaneous evaluation of multiple risk factors for HD patients’ mortality.

SI was developed as a modified logit on the basis of logistic regression models. This gave unique characteristics to SI similarly to outcome-propensity score [25]. Observational studies have many confounding background characteristics. To control various confounders simultaneously and evaluate them easily, it is necessary to replace the collection of confounders with one variable. SI summarizes various risk factors for death as confounders to a single adjusted composite risk factor. The association between exposures and outcomes can be adjusted for SI in place of individual risk factors. In this study, SI showed high accuracies of prediction of all-cause death of HD patients and PEW diagnosis. SI might be appropriate as a summary index for studies in which the effects of multiple exposures on these outcomes (i.e., all-cause death and PEW) are evaluated.

Determination of HD patients’ prognosis is effective for screening patients with a high risk of death and for evaluating patients’ condition. The capability of an index to predict patients’ risk of death clarifies which patients urgently need intervention. In this study, the predicted probability of death based on SI showed good agreement with the rate of observed all-cause death, and SI was associated with mortality in time-dependent Cox PHMs. Because SI is based on the prognosis of HD patients, can be easily measured, does not depend on the skill of examiners, and reflects HD patients’ specific characteristics, it may be a useful tool to identify high-risk HD patients and evaluate their condition. In clinical settings, after the screening, the patients need to be examined in detail.

CVDs are the leading causes of death in HD patients. From the Choices for Healthy Outcomes in Caring for ESRD study, a large percentage of incident dialysis patients were found to have the common risk factors for CVDs [26]. In HD patients, a lower BMI was not a predictor of incident CVDs but an independent risk factor for death after CVDs [4, 5]. PEW is one of the most important risk factors for death after CVDs [5]. SI included several factors (serum albumin, creatinine, and total cholesterol levels and BMI) listed in the ISRNM PEW criteria [3] and showed a high accuracy of PEW diagnosis. The coefficients of the variables in SI suggest that their changes toward PEW increased the risk of death. SI included serum phosphorus level. An elevated serum phosphorus level is associated with CVDs and increased mortality [20]. An increase in dietary protein intake has been shown to correlate with an increase in serum phosphorus level [27]. However, protein-intake restriction may lead to PEW. It has been reported that a prescribed reduction in phosphorus intake correlates with an increase in mortality [28]. And a decreased serum phosphorus level is also associated with increased mortality [20]. These results indicate the difficulty in keeping the balance between decreased serum phosphorus level and adequate nutrition by controlling excessive dietary intake.

There are specific associations between BMI and mortality in HD patients. First, BMI does not accurately reflect the body composition in HD patients. The protective effect of high BMI against mortality is limited to those patients with normal or high muscle mass [29]. The estimated rate of creatinine production has been used to assess the lean body mass in stable HD patients [30, 31]. Both BMI and serum creatinine level in SI can be used for the patients with an imbalance between BMI and muscle mass. Moreover, reverse epidemiology in HD patients has been reported to be a phenomenon due to the effects of age and inflammation on BMI [10, 11]. To adjust the interrelationships among BMI, age, and inflammation, the interactions among BMI, the square of BMI, age, and inflammation were evaluated, and finally BMI and age were included in SI. The coefficients of BMI and age in SI suggest that the patients’ prognosis worsened with decreasing in BMI and aging.

It has been reported that the relationship between BMI and mortality is U-shaped among HD patients aged <65 years and almost linear among those aged ≥65 years [11]. With the development of SI, it was observed that the coefficients of variables such as BMI and the square of BMI in SI changed depending on the study subpopulation (results not shown). Because the mean age of this study was more than 60 years, the quadratic function of BMI may have been approximated as a linear function of BMI. It was suggested that the appropriate model of the relationship between BMI and mortality may depend on the study population.

GNRI is composed of serum albumin level and BMI as continuous variables, and GNRI has been reported to reflect HD patients’ mortality [18]. CS is composed of categorical variables of serum albumin level, BMI and inflammation, and is associated with HD patients’ mortality [19]. However, the c-statistics of GNRI and CS were lower than those of single indices (serum albumin level and BMI) depending on the outcome. GNRI coefficients were originally based on the data from a population of veterans (99% of them were male, non-ESRD) [17, 32]. This suggests that an index with multiple variables may not always be accurate for the prediction of the outcome depending on the study population. On the other hand, although CS was developed on the basis of the HD-patient population, the cutoff values in CS may have not been appropriate for this study population. Thus, inappropriate cutoff values for a study may decrease the accuracy of CS for the prediction of the outcomes. These findings suggest that careful attention is required in using a risk index in a study population different from the population in which the risk index was developed. In this study, because SI also had this problem, stratification analysis for DM and vintage were examined to confirm the populations to which SI can be applied. The accuracy of SI in predicting death was higher than those of other indices in these populations examined. However, the problem was not completely eliminated, more studies are necessary to validate the usefulness of SI for various populations, such as those in various countries, and comorbid conditions.

In this study, some of the risk factors for death were included in SI. It was considered that these risk factors should be given priority in the population of this study. However, for example, DM as a cause of ESRD was not included in SI. A previous US DOPPS (DOPPS phases 1 and 2) also showed that DM as a cause of ESRD is not a statistically significant risk factor for one-year all-cause death [2]. On the other hand, another DOPPS showed that DM is commonly associated with mortality up to 5 years in patients from the US, Europe and Japan [33]. A significant difference in the prevalence of DM is observed in patients from the US, Europe and Japan [33]. These findings suggest that a factor such as DM is not always a statistically significant risk factor depending on the characteristics of the study population. Therefore, because each population of HD patients has peculiar important risk factors for death and their interrelationships, the main therapeutic targets may differ those of other populations. More international comparisons of patterns of HD therapy are important.

This study has several limitations. First, because of the observational nature of this study, the results may be biased by unmeasured confounders. Second, we were unable to examine the patients with missing data in this study, which might have caused selection bias. Third, the DOPPS datasets did not include enough nutritional data for assessing malnutrition, comorbid conditions and medications. We were unable to evaluate the effect of the differences in nutrition, comorbid conditions and medications on the risk of death. Further studies are needed to evaluate the relationship between these factors and SI. Fourth, because the DOPPS datasets did not include enough data to satisfy the requirements of other previously reported risk indices, SI could not be compared with those indices [34–39]. Fifth, the primary outcome of this study was all-cause death within one year; thus, we were unable to evaluate transplantation as an outcome. Sixth, the negative coefficient of serum phosphorus level in SI suggests a possibility that malnourished patients with low serum phosphorus levels may have a higher SI than malnourished patients with high serum phosphorus levels. However, because malnourished patients usually have low BMI and serum creatinine, albumin and total cholesterol levels, the errors caused by serum phosphorus levels may be minimized.

## Conclusions

The associations among many risk factors for death in HD patients are complex. This study showed a possibility that the simultaneous evaluation of multiple risk factors using SI can accurately assess patients’ prognosis and identify patients at increased risk of death.

## References

[1] Foley R, Murray A, Li S, Herzog C, McBean A, Eggers P, et al Chronic kidney disease and the risk for cardiovascular disease, renal replacement, and death in the United States Medicare population, 1998 to 1999. J Am Soc Nephrol. 2005;16(2):489–95. ASN.2004030203 [pii] 10.1681/ASN.2004030203 . 15590763

[2] Bradbury BD, Fissell RB, Albert JM, Anthony MS, Critchlow CW, Pisoni RL, et al Predictors of early mortality among incident US hemodialysis patients in the Dialysis Outcomes and Practice Patterns Study (DOPPS). Clin J Am Soc Nephrol. 2007;2(1):89–99. 10.2215/CJN.01170905 . 17699392

[3] Fouque D, Kalantar-Zadeh K, Kopple J, Cano N, Chauveau P, Cuppari L, et al A proposed nomenclature and diagnostic criteria for protein-energy wasting in acute and chronic kidney disease. Kidney Int. 2008;73(4):391–8. 5002585 [pii] 10.1038/sj.ki.5002585 . 18094682

[4] Beddhu S, Pappas LM, Ramkumar N, Samore MH. Malnutrition and atherosclerosis in dialysis patients. J Am Soc Nephrol. 2004;15(3):733–42. . 14978176 10.1097/01.asn.0000113319.57131.28

[5] Shoji T, Masakane I, Watanabe Y, Iseki K, Tsubakihara Y, Committee of Renal Data Registry, et al Elevated non-high-density lipoprotein cholesterol (non-HDL-C) predicts atherosclerotic cardiovascular events in hemodialysis patients. Clin J Am Soc Nephrol. 2011;6(5):1112–20. 10.2215/CJN.09961110 21511840 PMC3087778

[6] de Mutsert R, Grootendorst DC, Axelsson J, Boeschoten EW, Krediet RT, Dekker FW, et al Excess mortality due to interaction between protein-energy wasting, inflammation and cardiovascular disease in chronic dialysis patients. Nephrol Dial Transplant. 2008;23(9):2957–64. 10.1093/ndt/gfn167 . 18400817

[7] Gama-Axelsson T, Heimbürger O, Stenvinkel P, Bárány P, Lindholm B, Qureshi AR. Serum albumin as predictor of nutritional status in patients with ESRD. Clin J Am Soc Nephrol. 2012;7(9):1446–53. 10.2215/CJN.10251011 22723451 PMC3430958

[8] Herselman M, Esau N, Kruger JM, Labadarios D, Moosa MR. Relationship between body mass index and mortality in adults on maintenance hemodialysis: a systematic review. J Ren Nutr. 2010;20(5):281–92, 7 p following 92. 10.1053/j.jrn.2010.03.010 . 20580250

[9] Kalantar-Zadeh K, Block G, Humphreys MH, Kopple JD. Reverse epidemiology of cardiovascular risk factors in maintenance dialysis patients. Kidney Int. 2003;63(3):793–808. kid803 [pii] 10.1046/j.1523-1755.2003.00803.x . 12631061

[10] Leavey SF, McCullough K, Hecking E, Goodkin D, Port FK, Young EW. Body mass index and mortality in 'healthier' as compared with 'sicker' haemodialysis patients: results from the Dialysis Outcomes and Practice Patterns Study (DOPPS). Nephrol Dial Transplant. 2001;16(12):2386–94. . 11733631 10.1093/ndt/16.12.2386

[11] Hoogeveen EK, Halbesma N, Rothman KJ, Stijnen T, van Dijk S, Dekker FW, et al Obesity and mortality risk among younger dialysis patients. Clin J Am Soc Nephrol. 2012;7(2):280–8. 10.2215/CJN.05700611 22223612 PMC3280032

[12] Young E, Goodkin D, Mapes D, Port F, Keen M, Chen K, et al The Dialysis Outcomes and Practice Patterns Study (DOPPS): An international hemodialysis study. Kindey Int. 2000;57(74 [Suppl]):S74–S81.

[13] Pisoni RL, Gillespie BW, Dickinson DM, Chen K, Kutner MH, Wolfe RA. The Dialysis Outcomes and Practice Patterns Study (DOPPS): design, data elements, and methodology. Am J Kidney Dis. 2004;44(5 Suppl 2):7–15. . 15486868 10.1053/j.ajkd.2004.08.005

[14] Tentori F, Blayney MJ, Albert JM, Gillespie BW, Kerr PG, Bommer J, et al Mortality risk for dialysis patients with different levels of serum calcium, phosphorus, and PTH: the Dialysis Outcomes and Practice Patterns Study (DOPPS). Am J Kidney Dis. 2008;52(3):519–30. 10.1053/j.ajkd.2008.03.020 . 18514987

[15] Depner TA, Daugirdas JT. Equations for normalized protein catabolic rate based on two-point modeling of hemodialysis urea kinetics. J Am Soc Nephrol. 1996;7(5):780–5. . 8738814 10.1681/ASN.V75780

[16] Berrington de Gonzalez A, Hartge P, Cerhan JR, Flint AJ, Hannan L, MacInnis RJ, et al Body-mass index and mortality among 1.46 million white adults. N Engl J Med. 2010;363(23):2211–9. 10.1056/NEJMoa1000367 21121834 PMC3066051

[17] Bouillanne O, Morineau G, Dupont C, Coulombel I, Vincent JP, Nicolis I, et al Geriatric Nutritional Risk Index: a new index for evaluating at-risk elderly medical patients. Am J Clin Nutr. 2005;82(4):777–83. . 16210706 10.1093/ajcn/82.4.777

[18] Kobayashi I, Ishimura E, Kato Y, Okuno S, Yamamoto T, Yamakawa T, et al Geriatric Nutritional Risk Index, a simplified nutritional screening index, is a significant predictor of mortality in chronic dialysis patients. Nephrol Dial Transplant. 2010;25(10):3361–5. 10.1093/ndt/gfq211 . 20400447

[19] Takahashi R, Ito Y, Takahashi H, Ishii H, Kasuga H, Mizuno M, et al Combined values of serum albumin, C-reactive protein and body mass index at dialysis initiation accurately predicts long-term mortality. Am J Nephrol. 2012;36(2):136–43. 10.1159/000339940 . 22813921

[20] Fouque D, Roth H, Pelletier S, London GM, Hannedouche T, Jean G, et al Control of mineral metabolism and bone disease in haemodialysis patients: which optimal targets? Nephrol Dial Transplant. 2013;28(2):360–7. 10.1093/ndt/gfs404 . 23136211

[21] Palmer SC, Hayen A, Macaskill P, Pellegrini F, Craig JC, Elder GJ, et al Serum levels of phosphorus, parathyroid hormone, and calcium and risks of death and cardiovascular disease in individuals with chronic kidney disease: a systematic review and meta-analysis. JAMA. 2011;305(11):1119–27. 10.1001/jama.2011.308 . 21406649

[22] Lopes AA, Bragg-Gresham JL, Elder SJ, Ginsberg N, Goodkin DA, Pifer T, et al Independent and joint associations of nutritional status indicators with mortality risk among chronic hemodialysis patients in the Dialysis Outcomes and Practice Patterns Study (DOPPS). J Ren Nutr. 2010;20(4):224–34. 10.1053/j.jrn.2009.10.002 . 20060319

[23] Rocco M, Frankenfield D, Hopson S, McClellan W. Relationship between clinical performance measures and outcomes among patients receiving long-term hemodialysis. Ann Intern Med. 2006;145(7):512–9. 145/7/512 [pii]. . 17015869 10.7326/0003-4819-145-7-200610030-00009

[24] Plantinga L, Fink N, Jaar B, Sadler J, Levin N, Coresh J, et al Attainment of clinical performance targets and improvement in clinical outcomes and resource use in hemodialysis care: a prospective cohort study. BMC Health Serv Res. 2007;7:5 1472-6963-7-5 [pii] 10.1186/1472-6963-7-5 17212829 PMC1783649

[25] Rosenbaum P, Rubin D. The Central Role of the Propensity Score in Observational Studies for Causal Effects. Biometrika. 1983;70(1):41–55.

[26] Longenecker JC, Coresh J, Powe NR, Levey AS, Fink NE, Martin A, et al Traditional cardiovascular disease risk factors in dialysis patients compared with the general population: the CHOICE Study. J Am Soc Nephrol. 2002;13(7):1918–27. . 12089389 10.1097/01.asn.0000019641.41496.1e

[27] Kalantar-Zadeh K, Gutekunst L, Mehrotra R, Kovesdy CP, Bross R, Shinaberger CS, et al Understanding sources of dietary phosphorus in the treatment of patients with chronic kidney disease. Clin J Am Soc Nephrol. 2010;5(3):519–30. 10.2215/CJN.06080809 . 20093346

[28] Lynch KE, Lynch R, Curhan GC, Brunelli SM. Prescribed dietary phosphate restriction and survival among hemodialysis patients. Clin J Am Soc Nephrol. 2011;6(3):620–9. 10.2215/CJN.04620510 21148246 PMC3082422

[29] Beddhu S, Pappas LM, Ramkumar N, Samore M. Effects of body size and body composition on survival in hemodialysis patients. J Am Soc Nephrol. 2003;14(9):2366–72. . 12937315 10.1097/01.asn.0000083905.72794.e6

[30] Canaud B, Garred LJ, Argiles A, Flavier JL, Bouloux C, Mion C. Creatinine kinetic modelling: a simple and reliable tool for the assessment of protein nutritional status in haemodialysis patients. Nephrol Dial Transplant. 1995;10(8):1405–10. . 8538933

[31] Keshaviah PR, Nolph KD, Moore HL, Prowant B, Emerson PF, Meyer M, et al Lean body mass estimation by creatinine kinetics. J Am Soc Nephrol. 1994;4(7):1475–85. . 8161729 10.1681/ASN.V471475

[32] Perioperative total parenteral nutrition in surgical patients. The Veterans Affairs Total Parenteral Nutrition Cooperative Study Group. N Engl J Med. 1991;325(8):525–32. 10.1056/NEJM199108223250801 . 1906987

[33] Goodkin DA, Bragg-Gresham JL, Koenig KG, Wolfe RA, Akiba T, Andreucci VE, et al Association of comorbid conditions and mortality in hemodialysis patients in Europe, Japan, and the United States: the Dialysis Outcomes and Practice Patterns Study (DOPPS). J Am Soc Nephrol. 2003;14(12):3270–7. . 14638926 10.1097/01.asn.0000100127.54107.57

[34] Charlson ME, Pompei P, Ales KL, MacKenzie CR. A new method of classifying prognostic comorbidity in longitudinal studies: development and validation. J Chronic Dis. 1987;40(5):373–83. . 3558716 10.1016/0021-9681(87)90171-8

[35] Athienites NV, Miskulin DC, Fernandez G, Bunnapradist S, Simon G, Landa M, et al Comorbidity assessment in hemodialysis and peritoneal dialysis using the index of coexistent disease. Semin Dial. 2000;13(5):320–6. . 11014695 10.1046/j.1525-139x.2000.00095.x

[36] Miskulin D, Bragg-Gresham J, Gillespie BW, Tentori F, Pisoni RL, Tighiouart H, et al Key comorbid conditions that are predictive of survival among hemodialysis patients. Clin J Am Soc Nephrol. 2009;4(11):1818–26. 10.2215/CJN.00640109 19808231 PMC2774950

[37] Liu J, Huang Z, Gilbertson DT, Foley RN, Collins AJ. An improved comorbidity index for outcome analyses among dialysis patients. Kidney Int. 2010;77(2):141–51. 10.1038/ki.2009.413 . 19907414

[38] Chua HR, Lau T, Luo N, Ma V, Teo BW, Haroon S, et al Predicting first-year mortality in incident dialysis patients with end-stage renal disease—the UREA5 study. Blood Purif. 2014;37(2):85–92. 10.1159/000357640 . 24589505

[39] Moss AH, Ganjoo J, Sharma S, Gansor J, Senft S, Weaner B, et al Utility of the "surprise" question to identify dialysis patients with high mortality. Clin J Am Soc Nephrol. 2008;3(5):1379–84. 10.2215/CJN.00940208 18596118 PMC2518805

